# Double Baltazar Procedure for Repair of Gastric Leakage Post-Sleeve Gastrectomy from Two Sites: Case Report of New Surgical Technique

**DOI:** 10.1007/s11695-018-3241-9

**Published:** 2018-04-18

**Authors:** Hussein Mcheimeche, Samer H. Dbouk, Riad Saheli, Dany Lichaa, Louai S. Chalaby

**Affiliations:** Department of General Surgery, Al Zahraa University Hospital, Jnah, Beirut, Lebanon

**Keywords:** Obesity, Sleeve gastrectomy, Gastric leakage post-sleeve, Gastric fistula post-sleeve, Baltazar fistulo-jejunostomy, Double Baltazar procedure

## Abstract

**Background:**

Staple line leak is one of the most challenging complications following laparoscopic sleeve gastrectomy, with a rate reaching near 1%. Its management often implicates a multidisciplinary approach and experienced bariatric and metabolic surgeons. The literature is abundant on various approaches to treat single staple line leak with variable results. But what to do in front of an intra-op incidental finding of double gastric fistulae?

**Methods:**

In this article, we describe a new successful surgical treatment option of double Baltazar technique for a patient who was found to have two gastric fistulae post-sleeve gastrectomy. We aim to demonstrate that this approach is safe and effective and can help avoid major side effects of traditional treatment options for such complications.

**Results:**

The patient presented 20 days following a laparoscopic sleeve gastrectomy in a severe septic condition and was found to have a gastric leak. During surgical repair, unlike the usual single proximal fistula findings, another opening was identified more distally. Decision was made to proceed with a double fistulo-jejunostomy. It was a feasible technique, with no intra-op complications. Post-operatively, the patient had a successful recover, with no residual leak.

**Conclusions:**

Double Baltazar technique is a successful and feasible treatment option for patients presenting with two gastric fistulae following sleeve gastrectomy. This is the first case report describing this new technique, and its success should encourage more similar trials and avoid more aggressive surgical options such as total gastrectomy or gastric bypass.

## Introduction

Laparoscopic sleeve gastrectomy has become a widely accepted and effective bariatric procedure for the treatment of morbid obesity. It was initially introduced as the first step in a two-steps procedure of biliopancreatic diversion with duodenal switch in super obese patients, to decrease the surgical risk in this group of patients [1].

Sleeve gastrectomy has become one of the procedures of choice for management of morbid obesity, with high rates of resolution of obesity related comorbidities, average excess weight loss (reaching 70% after 3 years), and low complication rate. Patients also report minimal food intolerance in comparison to other bariatric surgeries such as laparoscopic adjustable gastric band [2, 3].

Gastric leak following laparoscopic sleeve gastrectomy remains a challenging complication with significant morbidity and prolonged hospital admissions. Most studies report a rate of leak of 1% when sleeve gastrectomy is performed by skilled and highly trained surgeons [4]. Its treatment should follow a multidisciplinary approach, as it may involve experienced surgeons and intensivists, infection control, nutritional support, experienced endoscopists, and interventional radiologists [5, 6].

Adequate drainage, broad spectrum antibiotics, and nutritional support are a priority in management of gastric leak. Surgical drainage may be the best drainage option since the collections are usually well-localized in the retro-gastric or sub-diaphragmatic area. During exploration, many options are available to close the staple line leak. Fistula site may be closed primarily if contamination is localized and local tissues are healthy. Re-sleeve of the fistula site may be an option in selected cases. The surgical closure of the fistula site may be deferred if there was evidence of hemodynamic instability or generalized peritonitis.

Conversion to Roux-en-Y gastric bypass is also a possible option to decrease the intra-gastric pressure and help in fistulae healing. However, this should be avoided in cases of severe peritonitis and hemodynamic instability.

The Baltazar procedure or the fistulo-jejunostomy is a well-described approach for the treatment of staple line leak post-sleeve gastrectomy, where the fistula site is anastomosed to a small bowel loop, and the biliary secretions are then diverted by a more distal jejunojejunostomy. However, this technique has only been adopted for single-site gastric leaks. What are the options available when dealing with a complicated gastric leak from two different sites instead of one?

## Case Presentation

**Fig. 1:**
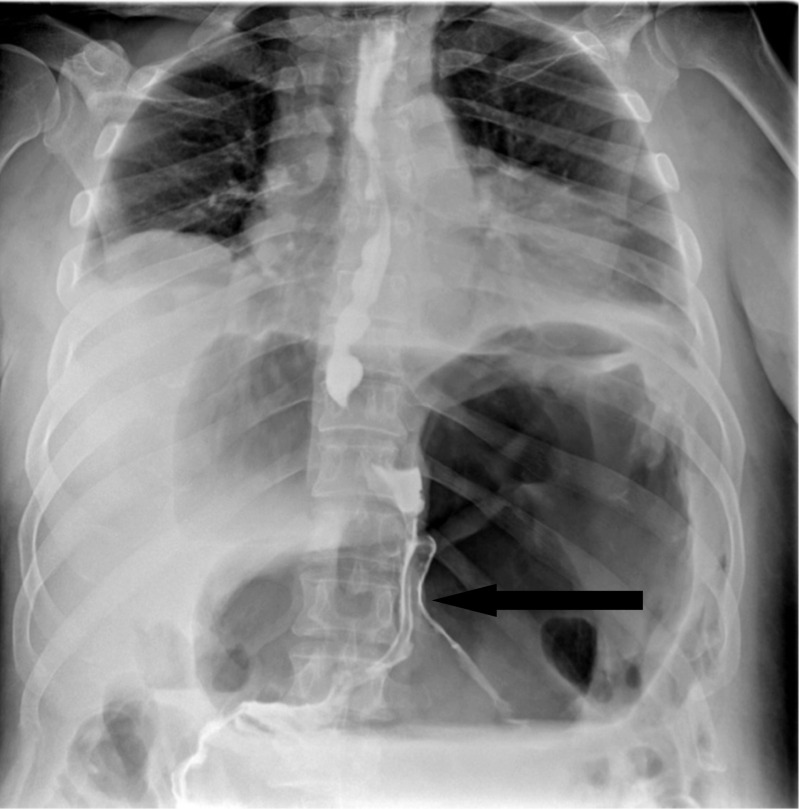
Upper GI showing the leak of gastrografine from the gastric fistula

**Fig. 2:**
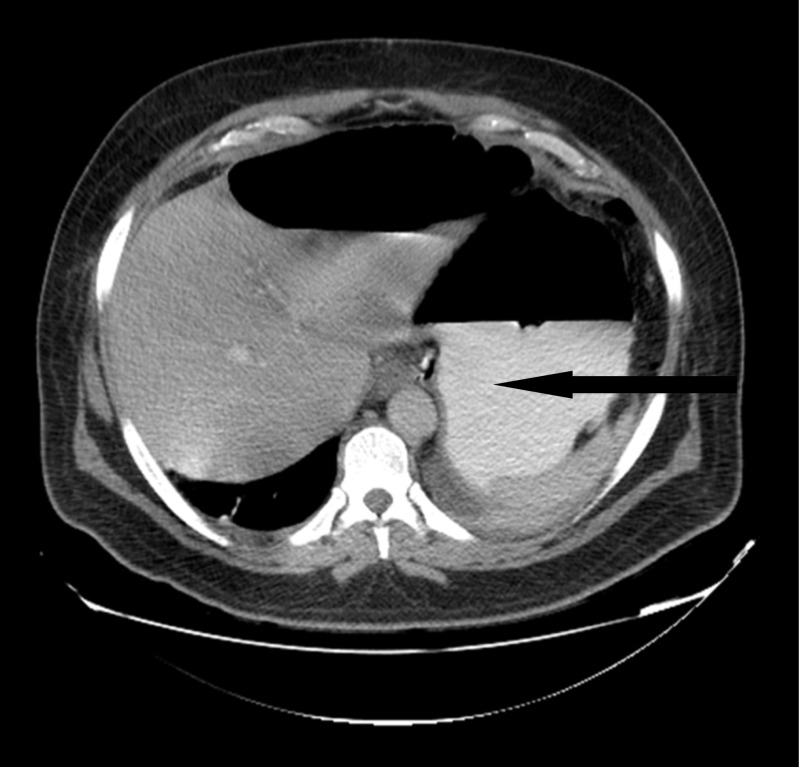
CT scan showed perigastric tube air-fluid collection

A 46-year-old gentleman, morbidly obese (BMI 57.4 kg/m^2^), was referred to our institute 20 days after a laparoscopic sleeve gastrectomy, complicated by gastric leak. On presentation, he was septic and in distress, tachycardic, and tachypneic. He was febrile and complaining of abdominal pain. Examination revealed a distended abdomen with diffuse tenderness and left basilar crackles on lung examination. Initial laboratory tests revealed elevated WBC and CRP. Upper GI series and CT scan of the abdomen showed evidence of contained gastric fistula with perigastric fluid collection (Figs. 1 and 2).

The patient was kept NPO, started on parenteral nutrition, intravenous antibiotics and was well-hydrated to control the sepsis. He underwent CT-guided drainage of the collection. One week post-drainage, upper GI series was repeated showed a well-drained gastric leak.

**Fig. 3:**
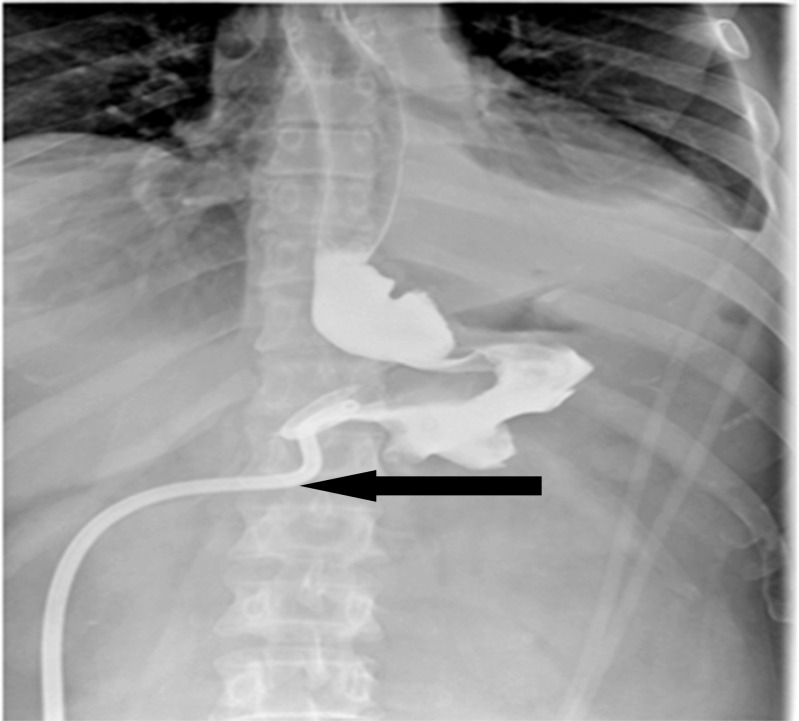
Upper GI showing well-drained gastric leak

After 10 days of stabilization, the patient showed marked improvement, became afebrile, and his WBC and CRP normalized, so a decision to undergo a Baltazar procedure was taken (Fig. 3).

### Surgical Procedure

After insufflation of the abdomen and insertion of trocars, lysis of loose adhesions was successfully done, in aim to uncover the gastric tube, which was covered with omental adhesions. The perigastric cavity was opened and well-irrigated, and with careful dissection, we unexpectedly identified two leak sites along the staple line, the first one was located 4 cm below the gastroesophageal junction, and the second one was located 6 cm away from the first fistula site. Unfortunately, stenting was not available at our institution.

**Fig. 4:**
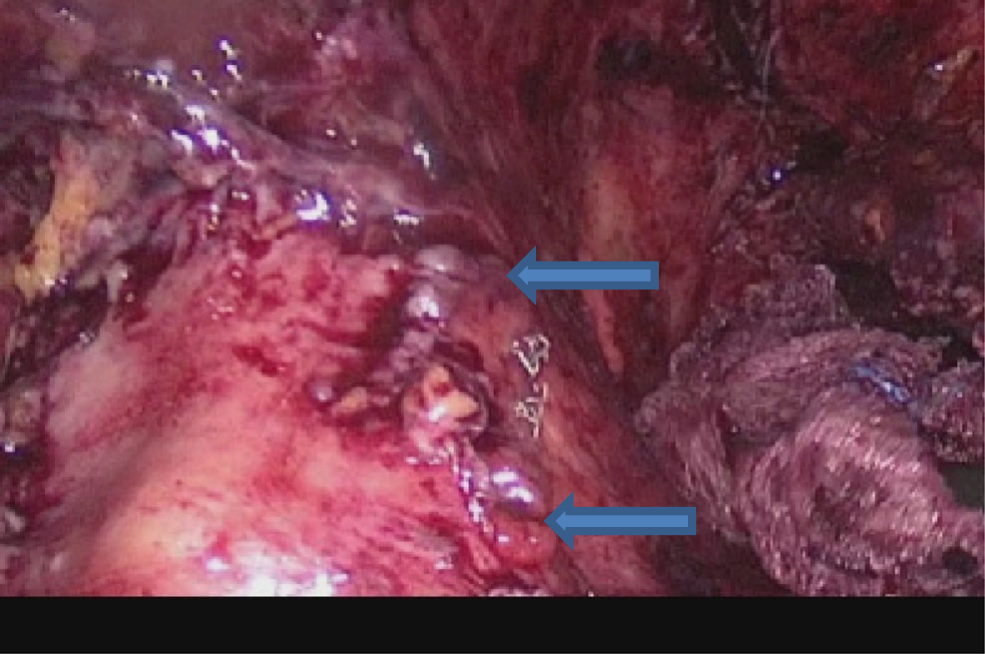
Laparoscopic picture showing the sites of the two gastric fistulae

After careful assessment, intra-op decision was made to attempt a new surgical technique: *double Baltazar procedure*. Two fistulo-jejunostomies were done with the same jejunal limb. The first fistulo-jejunostomy was done at the cephalic gastric fistula site with handsewn double-running 2/0 PDS suture. More distally to the created fistulo-jejunostomy, along the same jejunal loop, the second fistulo-jejunostomy was done using the same technique. Methylene blue test was done and showed no residual leak. Jejuno-jejunostomy (Brown anastomosis) was done after that to divert the biliary secretions and to protect the anastomotic sites. All quadrants of the abdomen were drained (Fig. 4).

## Results

The patient was kept NPO post-op, on antibiotics and TPN.

**Fig. 5:**
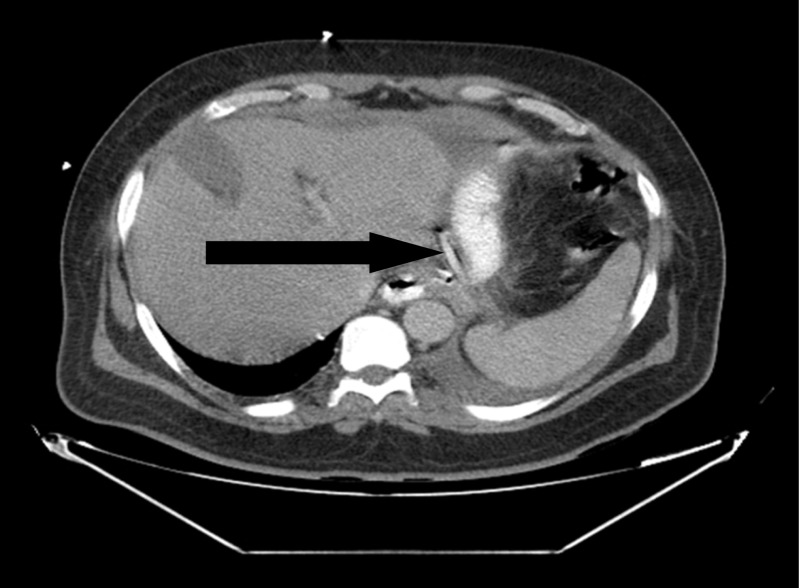
CT scan showing contrast in the hemovac tube (tiny leak of contrast from the 1st fistulo-jejunostomy)

On day 5 post-op, the patient underwent a CT scan with PO and IV contrast, which showed the presence of a small but well-drained leak (Fig. 5).

He was started on clear fluid diet on day 12 post-op, which was well-tolerated. On post-op day 14, upper GI was repeated and showed no extravasation of contrast material. Drains were removed, and the patient was planned for discharge.

Suddenly, he developed severe abdominal pain, with diffuse tenderness and guarding. WBC raised till 48,000 and CRP till 238. Abdominal CT scan showed evidence of acute acalculous cholecystitis. US-guided cholecystostomy was done, and the patient’s condition improved gradually.

**Fig. 6:**
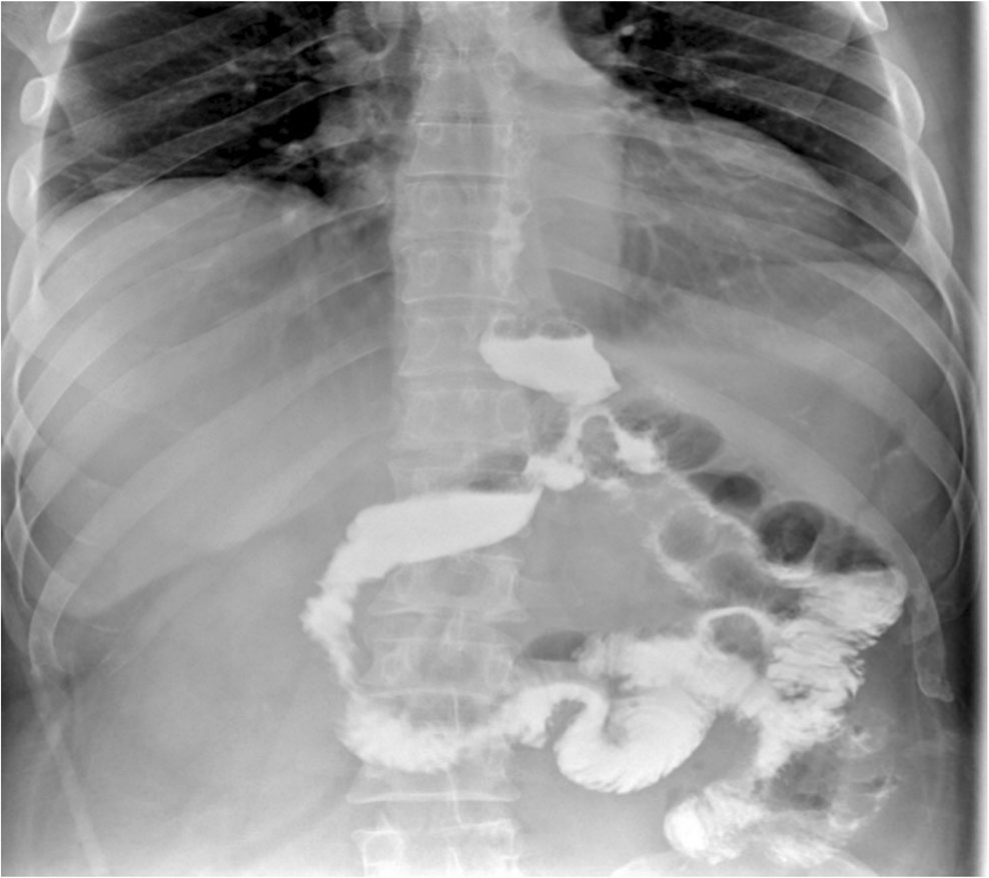
UGI showing complete healing of the fistulae

**Fig. 7:**
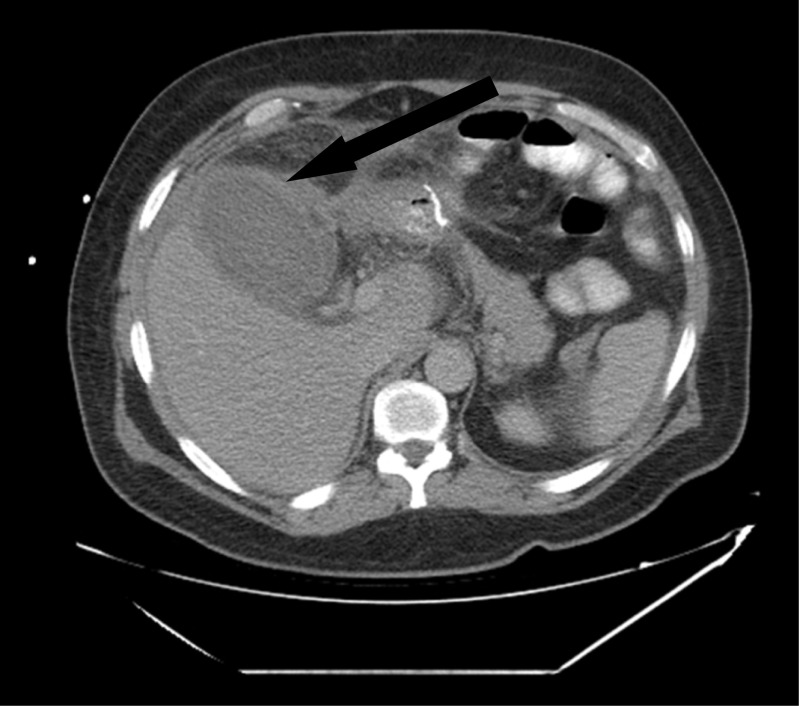
CT scan showing acute cholecystitis

On follow-up, the patient reported good recovery with an adequate weight loss. At 6 months, he has lost 80 kg, and his current BMI is at 33 kg/m^2^. He is tolerating well regular diet. A follow-up CT scan showed no residual leak or collections, with both fistulo-jejunostomies still patent (Figs. 6 and 7).

## Discussion

Sleeve gastrectomy is currently one of the most common bariatric procedures performed, with significant excess weight loss and resolution of comorbidities. Gastric leak remains a challenging complication. As mentioned earlier, gastric leaks post-sleeve gastrectomy should be managed in a multidisciplinary approach. The treatment of such complication ranges from peritoneal lavage with surgical or radiological drainage to primary repair of the fistula site, re-sleeve, stenting, or finally gastric bypass or total gastrectomy [7]. Recently, a technique for the treatment of gastric leaks, known as Baltazar procedure or the Roux-en-Y fistulo-jejunostomy has been shown to be safe and effective and is one of the valid surgical options for the treatment of gastric leaks post-laparoscopic sleeve gastrectomy [8–10].

In the presence of more than one leak site along the staple line, many would argue that a total gastrectomy is the only potentially successful treatment option. In this case, we described a successful approach, “the double Baltazar technique,” as a new surgical technique that would successfully treat a gastric leak from two sites, and thus avoid the need to perform total gastrectomy.

Our experience with the standard Baltazar procedure has been promising. We have had four patients at our institution who were treated with fisulo-jejunostomy. All of the four cases had excellent results as they were late fistulae, and the edges were suitable for repair. We consider Baltazar preocedure as the treatment of choice of late gastric leak post-sleeve gastrectomy. A jejuno-jejunal anastomosis (brown anastomosis) is usually performed as well to reduce the biliary reflux over the anastomotic site. Intervention should be performed after adequate stabilization and nutrition have been achieved. This period also allows for proper healing of the inflamed tissue so that they are suitable for surgical repair. We advise that such a procedure should be done between 1 and 2 months post-operatively to ensure a successful outcome.

## Conclusion

Surgical management of staple line leak post-laparoscopic sleeve gastrectomy has markedly evolved. Many options are currently described with variable outcomes and side effects. In this article, we described a new surgical technique (double Balthazar) as a promising option for the management of gastric leak post-sleeve gastrectomy from two gastric fistula sites. We believe that this technique, in hands of well-trained surgeons, can be a successful and a feasible option for treatment of this challenging complication with minimal side effects.
